# The Black Esophagus: A Rare Case Presentation

**DOI:** 10.7759/cureus.74989

**Published:** 2024-12-02

**Authors:** Archit Garg, Mehak Bassi, Arkady Broder

**Affiliations:** 1 Internal Medicine, Saint Peter’s University Hospital, New Brunswick, USA; 2 Gastroenterology and Hepatology, Saint Peter’s University Hospital, New Brunswick, USA

**Keywords:** acute esophageal necrosis, black esophagus, endoscopy, ischemia, necrotizing esophagitis, upper gastrointestinal bleeding

## Abstract

Acute esophageal necrosis (AEN), also known as black esophagus or Gurvits syndrome, is an uncommon endoscopic finding characterized by diffuse, circumferential, black discoloration of the esophagus that terminates at the gastroesophageal junction. The incidence of AEN has been reported to be 0-0.2% in autopsy series and up to 0.2% in observational studies. More common in elderly men, AEN usually presents with signs of upper gastrointestinal bleeding, such as hematemesis and melena, and is diagnosed by upper endoscopy. In severe cases, it can cause hemorrhagic shock, esophageal perforation, and mediastinitis. We present the case of a 78-year-old male with a history of diabetes mellitus, coronary artery disease, and peripheral arterial disease who presented with abdominal pain, obstipation, and vomiting for four days. He was found to have a small bowel obstruction secondary to incarcerated inguinal hernia and underwent hernia repair. The postoperative course was complicated by gastrointestinal bleeding requiring endoscopic examination, which revealed black discoloration of the esophagus, confirming the diagnosis of AEN. Management included aggressive fluid resuscitation, proton pump inhibitors, and nutritional support. Endoscopic interventions were performed to control bleeding. Physicians need to have a high index of suspicion for AEN in elderly patients with upper gastrointestinal bleeding due to the increased incidence in this age group. Early diagnosis and management can prevent complications and improve outcomes.

## Introduction

Acute necrotizing esophagitis, also known as black esophagus and acute esophageal necrosis (AEN), is a rare condition whose incidence has been reported to be less than 0.3% in endoscopic studies. First described by Goldenberg in 1990, AEN is usually caused by ischemic insult of the esophageal mucosa, reflux of corrosive acidic contents from the stomach, and esophageal mucosa barrier dysfunction [1,2]. It affects the elderly, with most patients presenting with signs of upper gastrointestinal bleeding [2]. Endoscopy is used to diagnose AEN, which presents as black mucosa with an abrupt transition to normal mucosa at the gastroesophageal junction. Usually localized to the distal esophagus, AEN commonly extends proximally to varying degrees [3]. However, milder cases may present with vague symptoms like chest pain, dysphagia, or heartburn and can be misdiagnosed as other conditions like reflux esophagitis. Since a definite diagnosis requires endoscopy, which is often performed in patients with significant upper gastrointestinal bleeding, this can lead to AEN being underdiagnosed. Complications include perforation, mediastinitis, stenosis, stricture formation, and overall increased mortality up to 32% [4]. Our case describes a 78-year-old man with uncontrolled diabetes and a long-standing coronary artery, found to have necrosis in the entire esophagus after the repair of an incarcerated hernia surgery. Gastroenterologists should know this rare entity so early treatment can be offered to prevent complications.

## Case presentation

A 78-year-old man presented to our emergency department with a four-day history of obstipation and vomiting. His medical history was significant for coronary artery disease (CAD), symptomatic peripheral artery disease (PAD), hyperlipidemia, hypertension, and type 2 diabetes mellitus (for which he was taking metformin 1000 mg twice daily, glyburide 2.5 mg daily, and sitagliptin 100 mg daily for 30 years). The patient was diagnosed with CAD eight years ago and underwent coronary artery bypass graft surgery followed by percutaneous coronary artery intervention five years later. He had been on aspirin 81 mg, clopidogrel 75 mg, and atorvastatin 40 mg daily after being diagnosed with CAD. He was also diagnosed with PAD seven years ago with an ankle-brachial index (ABI) of 0.4 (reference range: 1.0-1.4) confirmed with angiography of 70-80% common iliac artery stenosis on both the left and right sides. The patient underwent right femoral endarterectomy, right common iliac angioplasty with stent placement, and left common iliac angioplasty with stent placement. He reported occasional alcohol consumption (one to two glasses of wine in a month for more than 50 years) but no history of ingestion of caustic agents such as oven cleaners, drain openers, toilet cleaners, detergents, dishwashing agents, phenol, etc. He was found to have a small bowel obstruction secondary to an incarcerated inguinal hernia for which he underwent open bilateral inguinal hernia mesh repair. The patient remained hemodynamically stable since admission with minimal blood loss during surgery and no operative complications. 

On the third postoperative day, the patient had five episodes of maroon-colored bloody bowel movements with clots. On physical examination, he was altered and had cold extremities. Vitals showed hypotension and tachycardia as shown in Table 1. Laboratory investigations showed a drop in hemoglobin (Hb) from 10 gm/dL to 6.9 gm/dL (reference range: 13-17 gm/dL), an elevation in blood urea nitrogen (BUN) from 18 to 71 (reference range: 9-28 mg/dL), and a rise in serum creatinine from 1.26 mg/dL to 2.24 mg/dL (reference range: 0.66-1.25 mg/dL). Aggressive resuscitation with fluids and blood products was initiated. A pantoprazole 80 mg bolus followed by a pantoprazole drip at 8 mg/hr was started. Aspirin and clopidogrel (which were initially held for surgery and restarted postoperatively) were also discontinued. He was transferred to the intensive care unit, where he underwent emergent esophagogastroduodenoscopy (EGD).

**Table 1:** Vital signs

Vital Sign	Patient Value	Reference Range
Systolic blood pressure (mmHg)	90	90-120
Diastolic blood pressure (mmHg)	30	60-80
Heart rate (beats/min)	110	60-110
Respiratory rate (breaths/min)	13	16-Dec
Temperature (Celsius)	99	36.4-37.2
Oxygen saturation (%)	95	95-100

**Figure 1:**
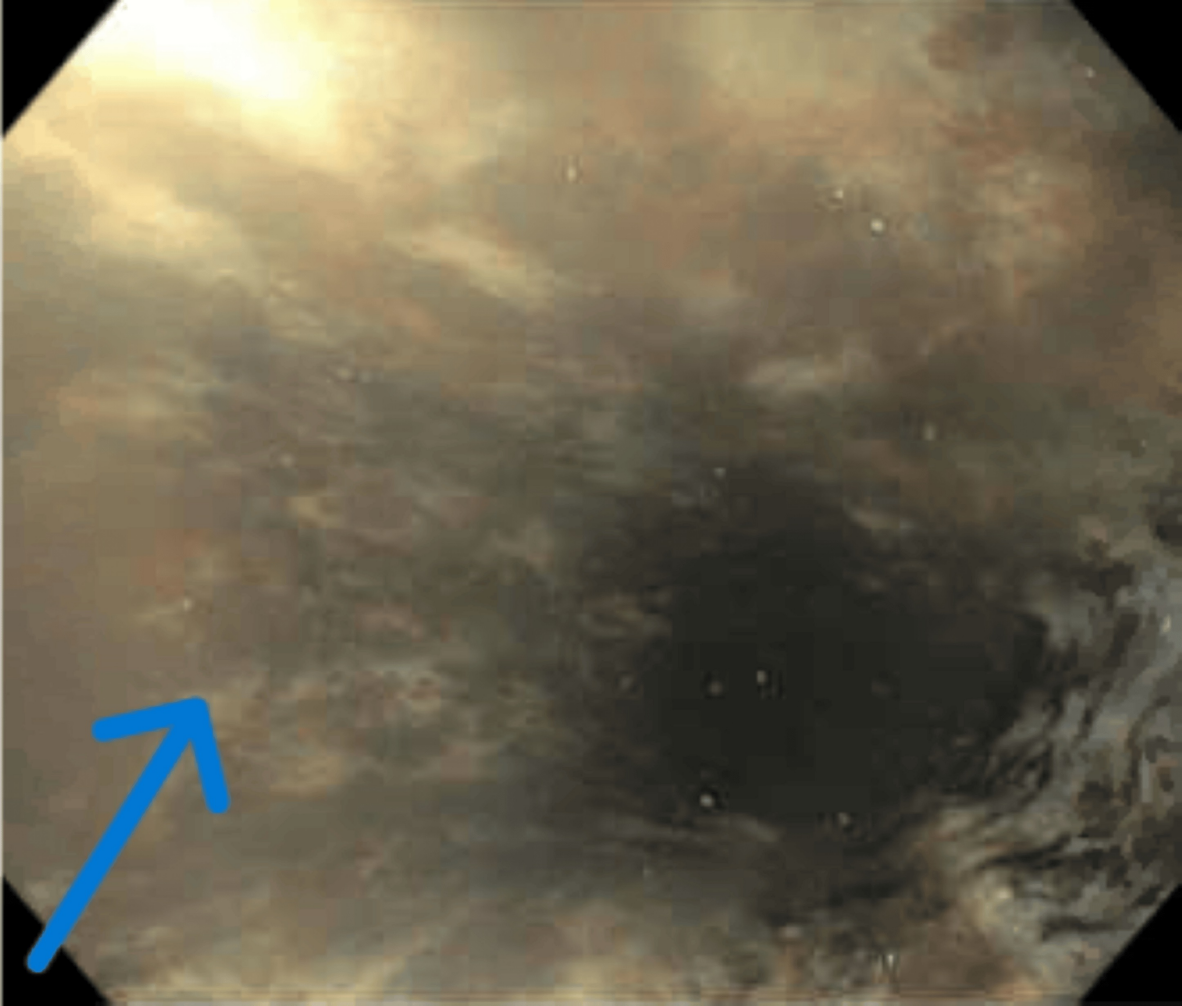
EGD showing circumferential necrosis in the upper middle part of the esophagus The arrow depicts necrosis with blackening, hence the name black esophagus EGD: esophagogastroduodenoscopy

**Figure 2:**
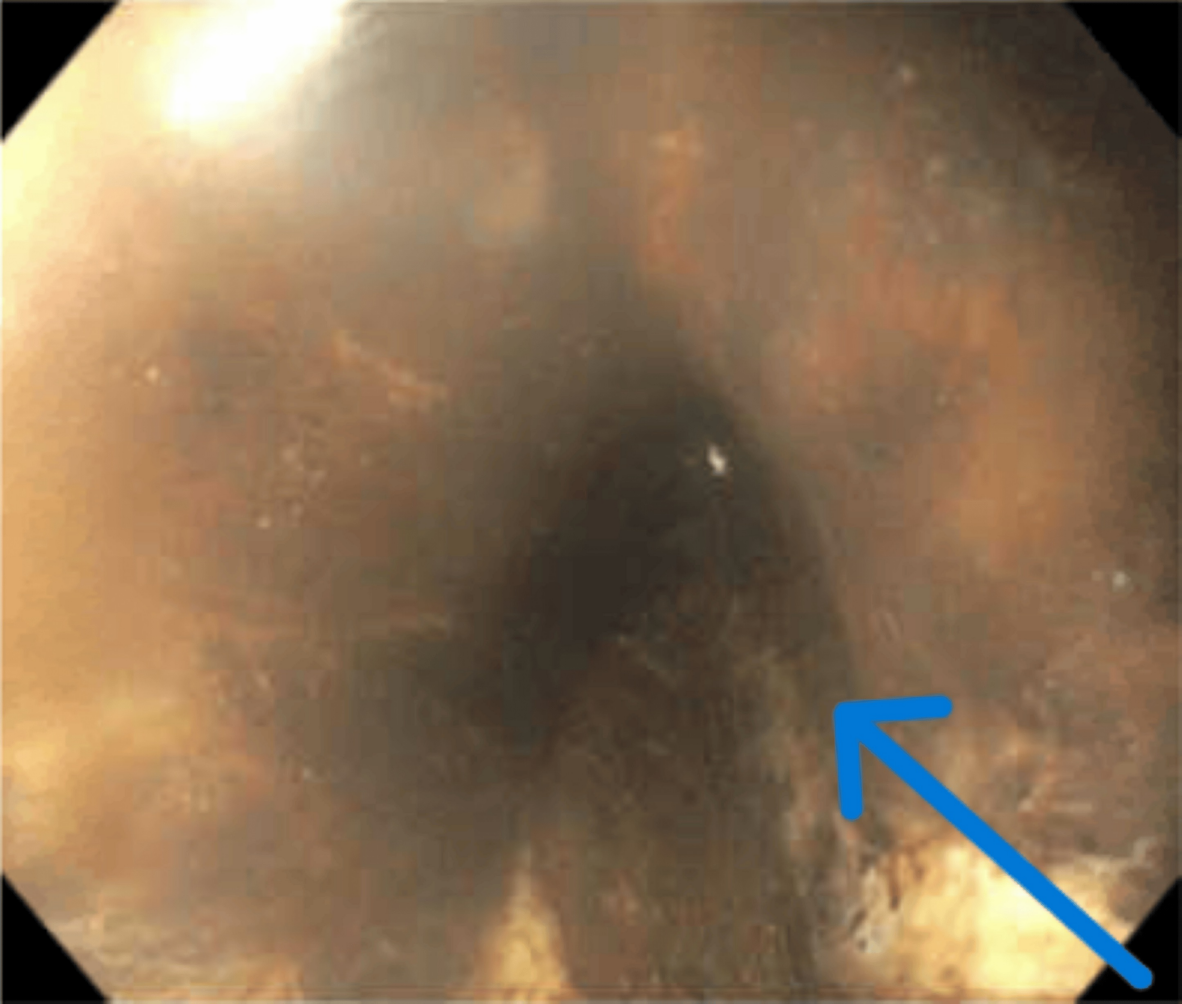
EGD showing circumferential necrosis in the middle third of the esophagus The arrow depicts necrosis all around the esophagus involving the complete mucosa EGD: esophagogastroduodenoscopy

**Figure 3:**
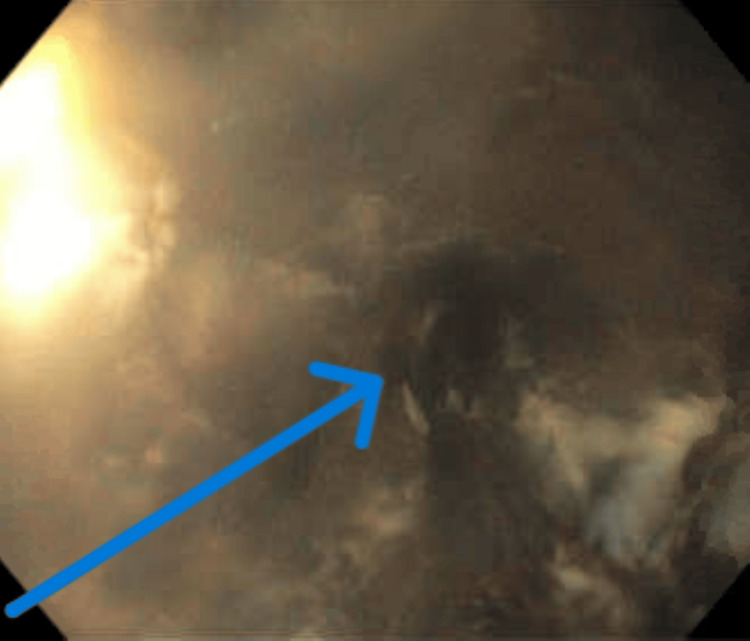
EGD showing circumferential necrosis in the middle lower third of the esophagus The arrow depicts the extension of necrosis which had extended from the upper middle to the lower third of the esophagus as the endoscope progressed through the esophagus EGD: esophagogastroduodenoscopy

**Figure 4:**
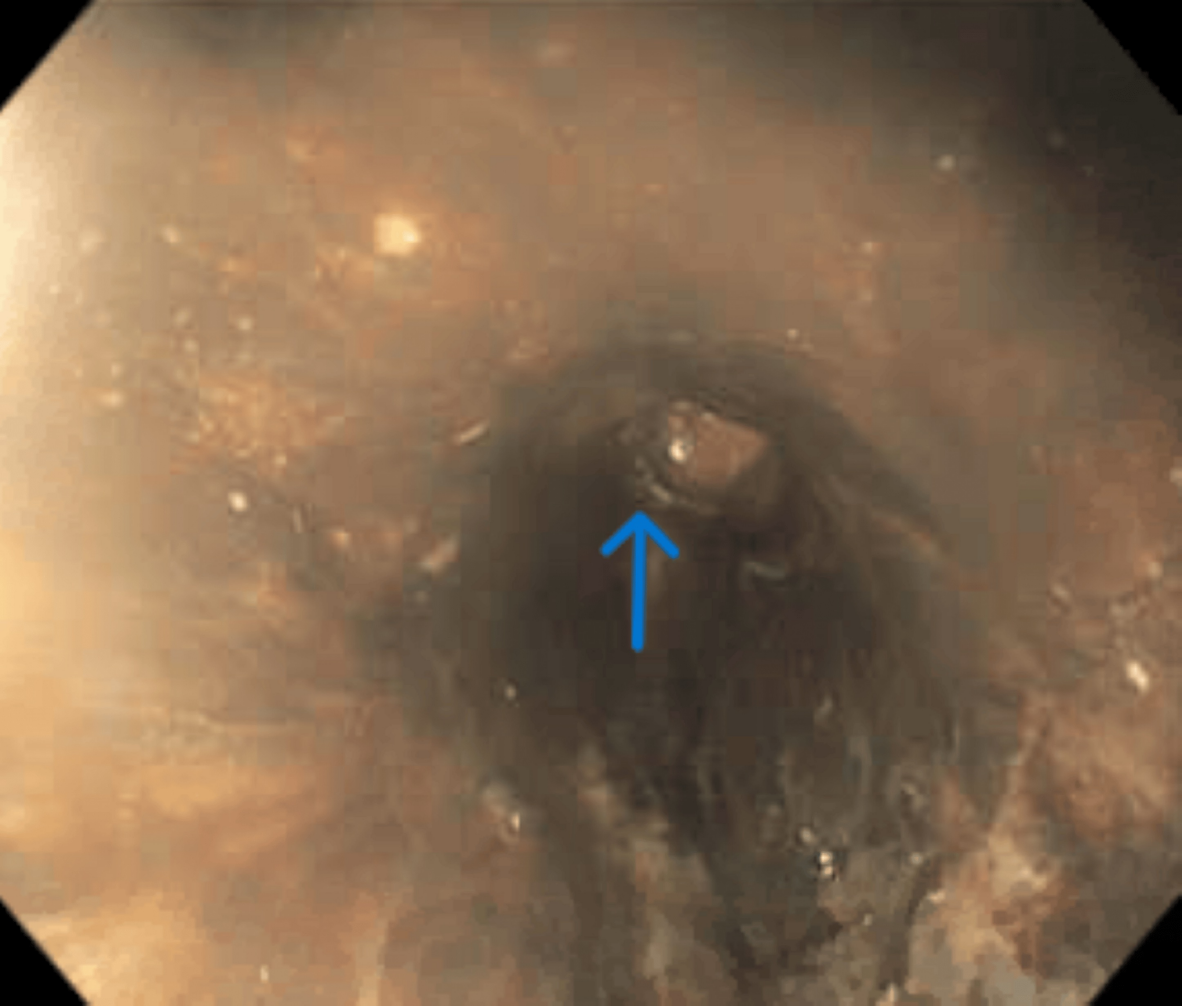
EGD showing circumferential necrosis extending to the lower third of the esophagus The arrows depict that the necrosis persisted all the way up to the lower segment of the esophagus till the gastroesophageal junction EGD: esophagogastroduodenoscopy

**Figure 5:**
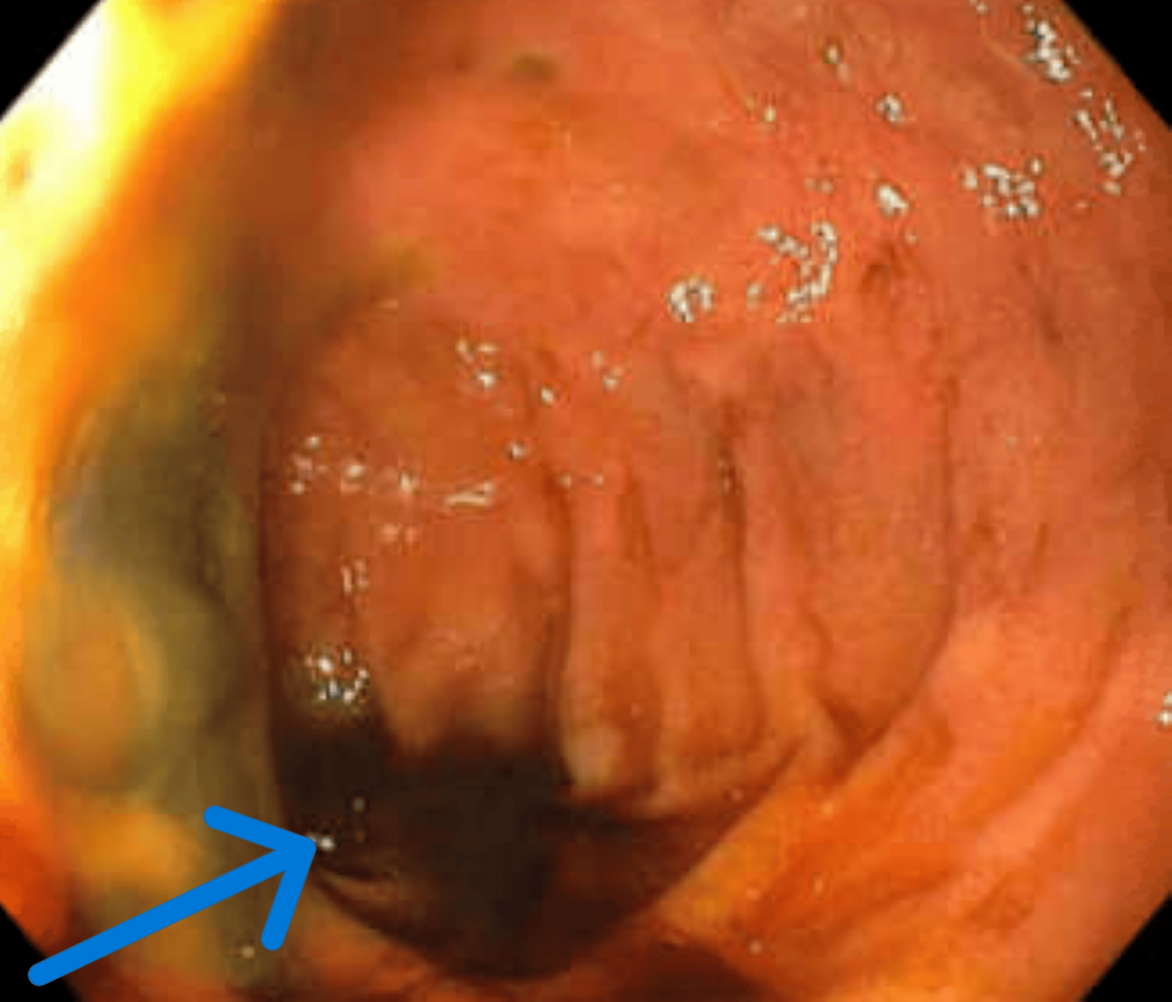
EGD showing the first part of the duodenum with necrotic debris The arrow depicts necrotic debris that passed down from the esophagus and stomach to the duodenum EGD: esophagogastroduodenoscopy

**Figure 6:**
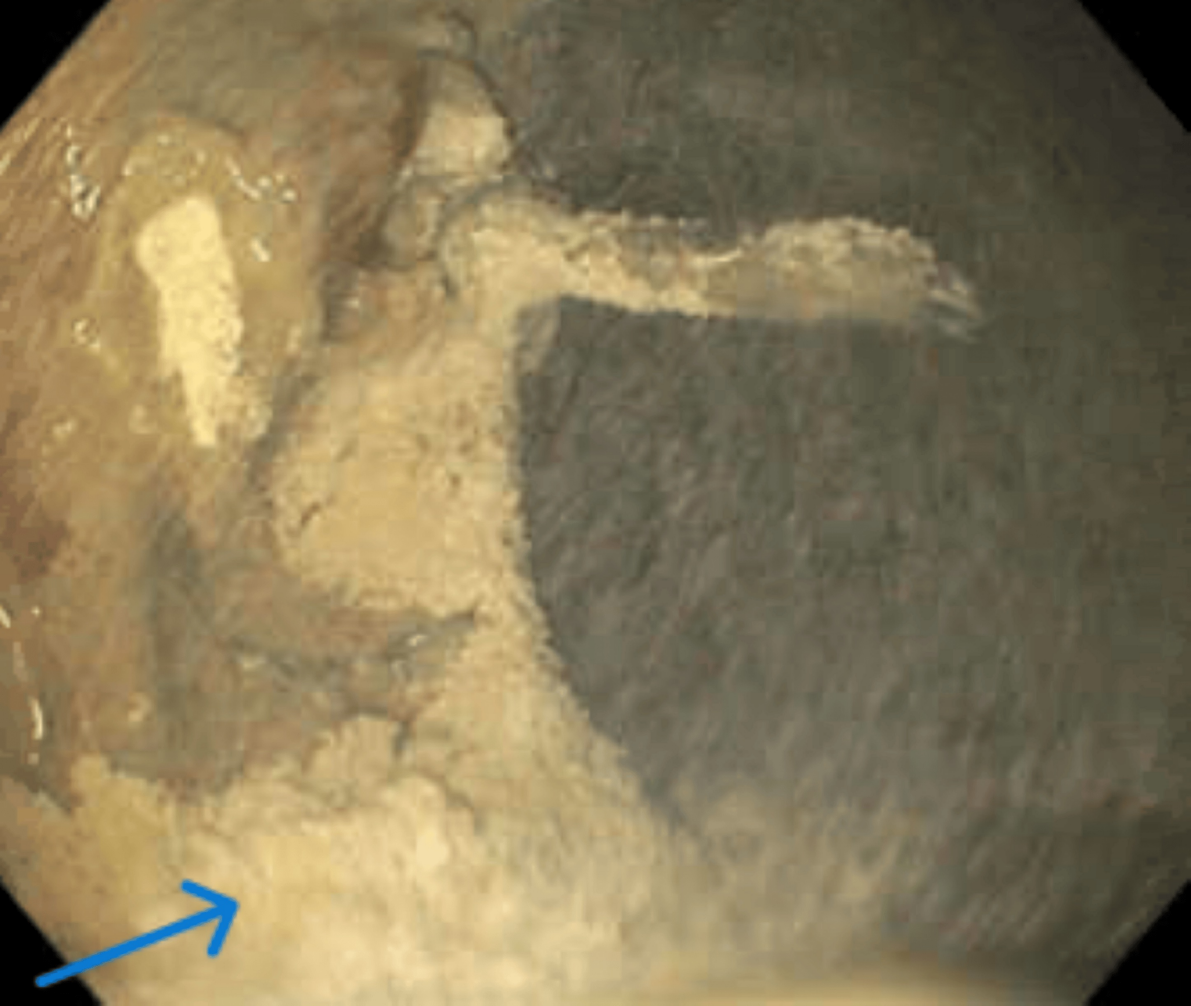
EGD showing the second part of the duodenum with necrotic debris and sloughed-off mucosa The arrow depicts sloughed-off mucosa with necrotic debris EGD: esophagogastroduodenoscopy

**Figure 7:**
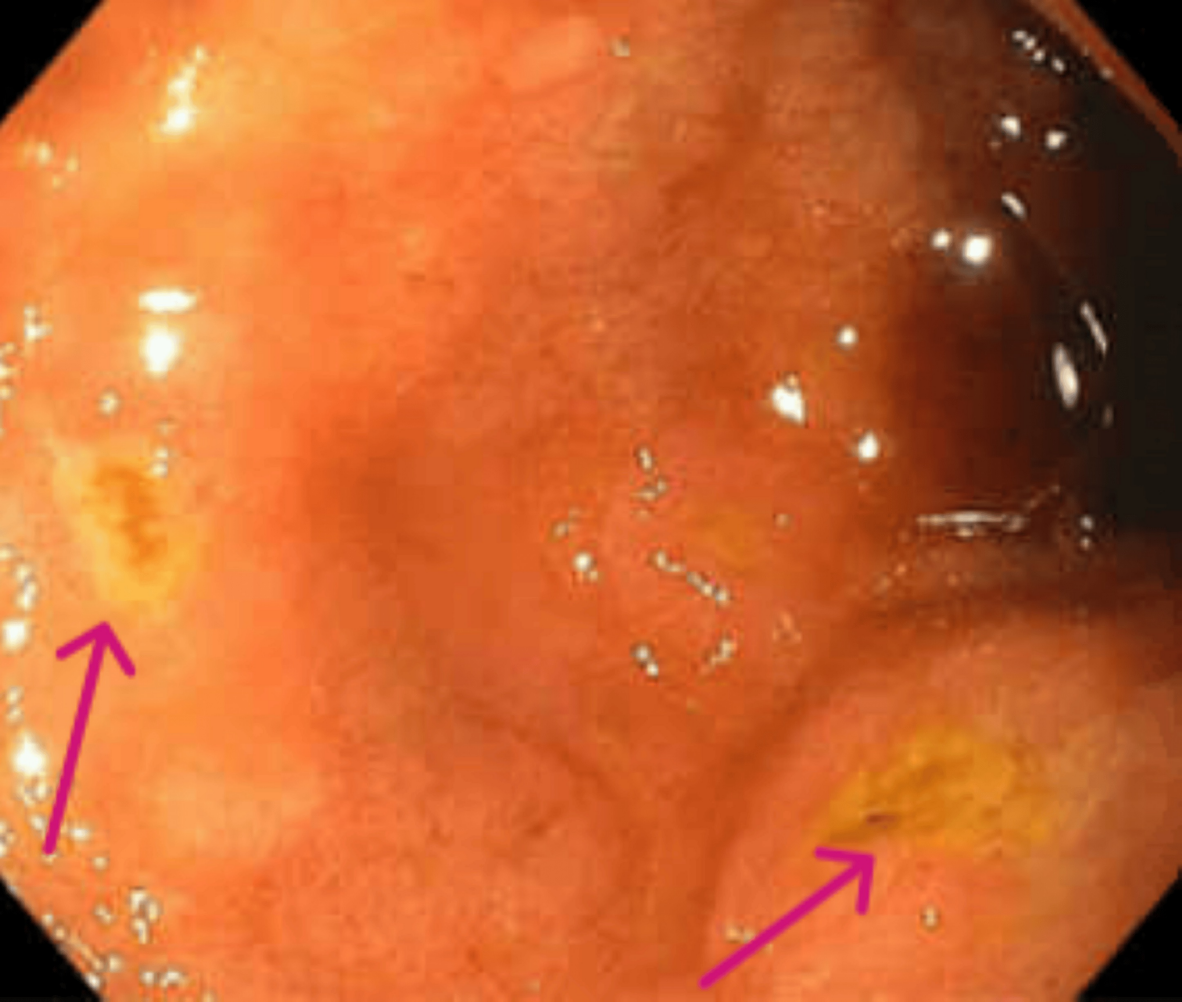
EGD showing ulcers in the duodenal bulb The two arrows depict superficial ulcers as seen in the duodenal bulb EGD: esophagogastroduodenoscopy

**Figure 8:**
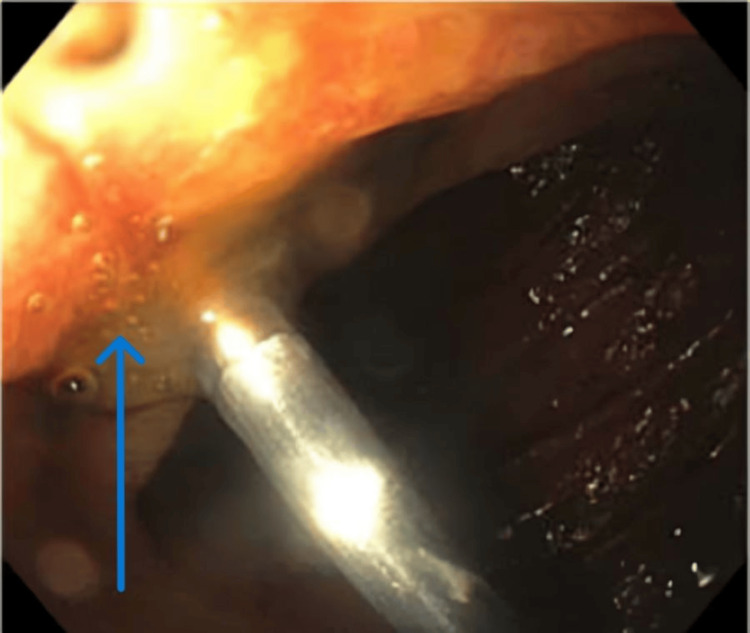
Clip applied to the ulcer in the second part of the duodenum The arrow depicts the application of the clip to the non-bleeding ulcer

EGD revealed a diffuse circumferential black appearance of the entire esophagus, suggesting AEN (Figures 1, 2, 3, 4 in order as the endoscope progressed from the mouth to the stomach). There was altered blood/coffee ground material in the stomach. The first and second parts of the duodenum showed necrotic debris with sloughed-off mucosa that might have been passed down from the esophagus and stomach (Figures 5, 6). Additional findings included ulcers in the duodenal bulb (Figure 7). One ulcer in the second part of the duodenum showed a non-bleeding visible vessel that was clipped (Figure 8). No bleeding was observed at the end of the procedure. With the patient’s history of diabetes, severe PAD, and CAD, surgical intervention for the incarcerated hernia might have acted as a trigger for AEN, and initiation of anticoagulants caused acute gastrointestinal bleeding.

The treatment included conservative management with fluids, blood transfusion (the patient received a total of five units of packed red blood cells), discontinuation of anticoagulants, and initiation of proton pump inhibitors (PPIs) (initially 80 mg pantoprazole IV bolus followed by a drip (8 mg/hr) for 72 hours and transitioned to 40 mg twice a day). The patient was initially kept nil per oral (NPO) till the patient was hemodynamically stable and there was no active bleeding. His diet progressed slowly as tolerated. The patient recovered well with stabilization of Hb and tolerating diet. Fifteen days after the bleeding episode, he was discharged home on daily PPI.

## Discussion

AEN is a severe condition characterized by the rapid and extensive death of esophageal tissue. It usually presents with diffuse involvement of the esophagus, with the distal esophagus being involved in 97% of cases [1,4]. It occurs four times more commonly in men, and peak incidence occurs in the elderly more commonly in the sixth decade of life [2,5]. Its incidence is less than 3%, an underestimation due to the transitory nature of the inciting trigger with quick mucosa recovery [6].

The exact etiology is not always clear; AEN is suggested to have multifactorial pathophysiology, including hypoperfusion causing ischemia of the esophagus from vascular insult, esophageal injury from reflux of corrosive gastric contents, and protective mucosal barrier disruption [1,2,5,6]. Ischemic insult occurs in chronic conditions like CAD (low-flow state) or hypotension in combination with an acute event such as surgery or gastric outlet obstruction triggering esophageal necrosis [7]. Reports have shown an association between shock and the development of AEN, even in young individuals [8,9]. Malnutrition has been found in as many as 40% of patients who experienced AEN [10,11]. Other risk factors predisposing to AEN include diabetes, CAD, hypertension, malignancy, alcoholism, gastric outlet obstruction, and acid reflux [2,4]. In our patient, several risk factors, including old age, male sex, CAD, and diabetes, along with the trigger in the form of hernia repair surgery, produced an ischemic insult causing mucosal barrier disruption of the esophagus, resulting in AEN. 

The most common clinical presentation of AEN, with an incidence of up to 80%, is gastrointestinal bleeding, hematemesis, or melena [12]. However, sometimes people can be asymptomatic and diagnosed with AEN incidentally on EGD. Diagnosis is made through EGD, which shows full-thickness circumferential blackening of the esophagus with clear-cut demarcation at the gastroesophageal junction [1-4]. Additional findings include coffee ground material in the stomach, duodenal ulcers or erosions, and gastric outlet obstruction [13]. In our patient, esophageal blackening, coffee ground material in the stomach, and duodenal ulcers were present. Histological examination shows necrotic changes in the mucosa extending up to the muscularis propria, leukocytic infiltration, and fibrinous deposition [7].

The most common complication of AEN is perforation. This can lead to mediastinitis and death. Other complications include esophageal strictures, stenosis, and gastric outlet obstruction [7]. Sepsis can occur in AEN, posing a substantial risk, especially in immunocompromised individuals [8,9]. The prognosis of acute esophageal necrosis (AEN) is poor, with a mortality rate reported to range from 32% to 43%, according to various studies [2,3]. The mortality risk is significantly associated with the presence of complications, such as perforation or sepsis. As such, timely diagnosis and aggressive management influence outcomes [4]. Management of AEN is multifaceted and aims to address the necrotic esophageal tissue and the contributing factors. Supportive measures, including nutritional support, intravenous fluids, blood products, proton pump inhibitors, or histamine receptor blockers, are vital in improving the patient's overall health [11]. Early endoscopic evaluation is essential for diagnosis. In addition, repeat endoscopy is important to evaluate the extent of mucosal healing and resolution of AEN and to determine the presence of long-term complications of AEN like stenosis or strictures. Therapeutic interventions such as dilation, stenting, or sclerotherapy to manage complications like strictures or bleeding may be required [2,4]. In severe cases, surgical intervention, including esophagectomy, may be considered [10].

There has been increased awareness among clinicians about the association of AEN with chronic immunosuppression and critical illness, putting the older population at increased risk for AEN. Schizas et al. in their systematic review in 2020 reported an increased incidence of surgical and endoscopic intervention over the years for the management of AEN [12]. However, there was no significant change in the outcome. They reported that the overall mortality rate was 29.9% despite intervention. This warrants further research to better understand the pathophysiology, associated risk factors, and optimum treatment options for AEN. Moreover, it also becomes pertinent to have AEN as a differential diagnosis in elderly patients with multiple comorbidities who present with upper gastrointestinal bleeding or atypical symptoms like epigastric/chest pain, nausea, vomiting, or dysphagia. 

## Conclusions

AEN is an uncommon cause of upper gastrointestinal bleeding, but incidence will likely increase as the elderly population increases. AEN should be considered as a differential in the case of upper gastrointestinal bleeding in elderly patients with multiple comorbidities and chronic immunosuppression. Diagnosis requires a high degree of suspicion, early EGD, and prompt resuscitation with fluids, pantoprazole, and blood transfusion. Without timely intervention, AEN has a poor prognosis; hence, time is of the essence. It is therefore important for clinicians to keep AEN as a differential, which, if not timely diagnosed, can have fatal outcomes.
